# A c-Jun N-terminal kinase inhibitor, JNK-IN-8, sensitizes triple negative breast cancer cells to lapatinib

**DOI:** 10.18632/oncotarget.20581

**Published:** 2017-08-24

**Authors:** Nancy D. Ebelt, Tamer S. Kaoud, Ramakrishna Edupuganti, Sabrina Van Ravenstein, Kevin N. Dalby, Carla L. Van Den Berg

**Affiliations:** ^1^ Institute of Cellular & Molecular Biology, University of Texas at Austin, Dell Pediatric Research Institute, Austin, TX 78723, USA; ^2^ Division of Chemical Biology and Medicinal Chemistry, College of Pharmacy, University of Texas at Austin, Austin, TX 78712, USA; ^3^ Division of Pharmacology & Toxicology, College of Pharmacy, University of Texas at Austin, Dell Pediatric Research Institute, Austin, TX 78723, USA; ^4^ Department of Medicinal Chemistry, Faculty of Pharmacy, Minia University, El-Minia 61519, Egypt

**Keywords:** triple negative breast cancer, lapatinib, JNK, oxidative stress, antioxidant

## Abstract

Triple negative breast cancers (TNBC) have poor prognosis compared to other breast cancer subtypes and represent 15-20% of breast cancers diagnosed. Unique targets and new molecularly-targeted therapies are urgently needed for this subtype. Despite high expression of Epidermal Growth Factor Receptor, inhibitors such as lapatinib have not shown therapeutic efficacy in TNBC patients. Herein, we report that treatment with the covalent JNK inhibitor, JNK-IN-8, synergizes with lapatinib to cause cell death, while these compounds as single agents have little effect. The combination significantly increases survival of mice bearing xenografts of MDA-MB-231 human TNBC cells. Our studies demonstrate that lapatinib treatment increases c-Jun and JNK phosphorylation indicating a mechanism of resistance. Combined, these compounds significantly reduce transcriptional activity of Nuclear Factor kappa B, Activating Protein 1, and Nuclear factor erythroid 2-Related Factor 2. As master regulators of antioxidant response, their decreased activity induces a 10-fold increase in reactive oxygen species that is cytotoxic, and is rescued by addition of exogenous antioxidants. Over expression of p65 or Nrf2 also significantly rescues viability during JNK-IN-8 and lapatinib treatment. Further studies combining JNK-IN-8 and lapatinib may reveal a benefit for patients with TNBC, fulfilling a critical medical need.

## INTRODUCTION

Triple negative breast cancers (TNBC) represent 15-20% of all breast cancers diagnosed in the U.S. [1, 2]. This tumor type (encompassing basal-like-1 and −2, immunomodulatory, mesenchymal, mesenchymal stem-like, luminal androgen receptor or unspecified) has very high rates of tumor recurrence and poor prognosis compared to other breast cancer subtypes [3–6]. Treatment of these tumors is difficult due to a lack of targetable receptors such as estrogen receptor and Human Epidermal growth factor Receptor (HER) 2, and although TNBCs initially respond well to chemotherapy, they exhibit high relapse rates [7, 8]. In light of the shortcomings of chemotherapy, the discovery of potent, molecularly-targeted therapies for TNBC is of great interest and urgently needed.

Many TNBCs overexpress EGFR (HER1), whose expression level correlates with poor prognosis [9], however, clinical trials testing EGFR inhibitors indicate that they are ineffective as single agents [10, 11]. Using the EGFR inhibitor gefitinib, immunohistochemical (IHC) analysis of tumors showed that while EGFR phosphorylation was inhibited, phospho-Akt and Ki67 remained high [10]. Lapatinib, despite inhibiting both EGFR and HER2, also lacks efficacy in treating TNBC [12], and only minimally inhibits proliferation of TNBC cell lines [13]. While resistance mechanisms have been reported for anti-EGFR/HER2 therapies in HER2-overexpressing breast cancer [14, 15] and other cancers [16, 17], the reason for the lack of efficacy of EGFR and EGFR/HER2 inhibitors in TNBC remains unknown.

Recent studies have shown that lapatinib induces cytotoxic oxidative stress in cells sensitive to EGFR/HER2 inhibition [18], and another group reversed lapatinib resistance with the addition of a reactive oxygen species (ROS)-inducing compound [19]. In tumors that are innately resistant to EGFR/HER2 blockade, such as TNBC, it is unknown how lapatinib might affect oxidative stress levels. As breast and other cancers are more sensitive to increased oxidative stress than normal tissues, compounds that would promote cytotoxic levels of ROS in tumors are currently being explored [20, 21].

Downstream of HER signaling, c-Jun N-terminal kinases (JNKs) convey responses from HER stimuli, and also merge signaling from other growth factor receptors, inflammatory cytokines, and other extracellular stress stimuli [22–25]. In breast cancer, phosphorylated JNK significantly correlates with EGFR expression, positivity for cytokeratins, and the triple negative phenotype [26]. Downstream of JNK, increased phosphorylation of c-Jun at JNK specific sites correlates with increased invasiveness and angiogenesis [27], as well as with distant metastasis and the presence of HER family receptors [28]. Isoform specific functions of JNK have been described. JNK2 correlates with decreased disease-free survival in breast cancer patients diagnosed with the Basal-like subtype [29], increases epithelial to mesenchymal transition (EMT) [30], and JNK2 knockdown reduces lung metastasis of mammary cancer cells [24]. JNK1 specifically functions in insulin response [31], epithelial to mesenchymal transition (EMT) [32], and regulation of certain tumor suppressors [33]. These data indicate that inhibition of JNK activity may be beneficial to the treatment of TNBC as well as invasive breast cancer.

To improve upon poorly selective, competitive JNK inhibitors, irreversible inhibitors of JNK were recently developed. Of these, JNK-IN-8 was found to be the most selective with high affinity for all three JNK isoforms. JNK-IN-8 covalently binds Cys116 in the catalytic sites of both JNK1 and JNK2 and potently inhibits phosphorylation of c-Jun at Ser63 in cells [34]. This compound is far more selective for JNK than SP600125 which is frequently used to study JNK functions [35].

Herein, we show that JNK-IN-8 and lapatinib synergistically decrease cell viability in human TNBC cell lines by promoting apoptosis. Similar results are seen in human xenograft tumors wherein the combination of lapatinib and JNK-IN-8 significantly lengthens the time to reach maximum tumor growth. The transcriptional activities of Nuclear Factor kappa B (NFκB), Activating Protein 1 (AP-1), and Nuclear factor erythroid 2-Related Factor 2 (Nrf2) in cells are significantly decreased by combination treatment with JNK-IN-8 and lapatinib, and oxidative stress is dramatically increased. Addition of ROS scavengers rescues cell death and blocks accumulation of reactive oxygen species resulting from exposure to lapatinib and JNK-IN-8 combination. Overexpression of the RelA subunit of NFκB or Nrf2 also significantly rescues viability after the combination treatment. These data indicate that lapatinib and JNK-IN-8 synergize to inhibit an antioxidant response leading to cytotoxic accumulation of oxidative stress.

## RESULTS

### JNK-IN-8 and lapatinib synergize to cause TNBC cell death

**Figure 1:**
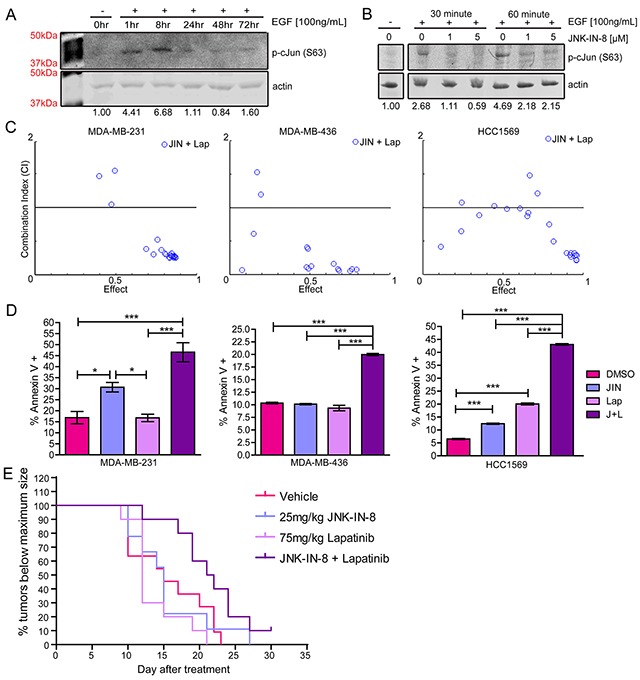
JNK-IN-8 and Lapatinib Synergize to Cause Cell Death in Triple-Negative Breast Cancer Cell Lines **(A)** MDA-MB-231 cells were serum-starved overnight and lysed (0hr) or stimulated with EGF for 1, 8, 24, 48, and 72 hours. Western blots of serine 63 phosphorylated c-Jun are shown with band densitometries beneath. **(B)** MDA-MB-231 cells were pre-treated for 3 hours with vehicle, 1μM, or 5μM JNK-IN-8 (JIN) and then stimulated (+) with EGF for 30 or 60 minutes as indicated. Western blots of phospho-c-Jun (Ser63) are shown with band densitometries beneath. **(C)** Combination Indexes (CI) for various concentrations of JIN and lapatinib (Lap) were calculated using CompuSyn software. Effect values represent percent decreased viability by MTT assay at 72 hours. Points below 1.0 CI (median line) are synergistic. **(D)** Annexin V positive cells were measured by flow cytometry. MDA-MB-231: JIN 5μM, Lap 3μM. MDA-MB-436: JIN 4μM, Lap 7μM. HCC1569: 3μM JIN, 1μM Lap. **(E)** Mice were injected orthotopically with MDA-MB-231cells and treated with vehicle, 25mg/kg JNK-IN-8 and/or 75mg/kg lapatinib. Time to maximum tumor size was recorded for each group starting at the time of the first treatment and presented as a Kaplan-Meier curve.

To determine the effectiveness of JNK-IN-8 in human TNBC cell lines, we tested its ability to inhibit EGF-induced JNK activity using the MDA-MB-231 cell line. JNK phosphorylated c-Jun shortly after EGF stimulation, peaking around 8 hours (Figure 1A). JNK-IN-8 at 1μM reduced c-Jun phosphorylation by 60% and 55% after 30 or 60 minutes of EGF stimulation, respectively. JNK-IN-8 at 5μM, inhibited phosphorylated c-Jun by 80% and 55% after 30 and 60 minutes of EGF stimulation (Figure 1B).

JNK-IN-8 covalently binds JNK1 and JNK2 at cysteine 116 [34]. To address whether JNK-IN-8 requires the cysteine residue to bind and inhibit JNK1 or JNK2, HEK293-T cells were used to overexpress wildtype (WT) or Cys116Ser mutant (MUT) JNK1 and JNK2 plasmids. Consistent with the requirement for cysteine 116 for JNK-IN-8 binding and inhibition, expression of both MUT JNK plasmids rescues c-Jun phosphorylation in JNK-IN-8 alone and JNK-IN-8 and lapatinib combination samples compared to cells expressing the WT plasmids (Supplementary Figure 1A). Both WT and MUT JNK isoforms were FLAG tagged and could be detected separately from endogenous JNK using a FLAG directed antibody. Western blots show that in cells where WT plasmids were overexpressed, presence of JNK-IN-8 results in a slightly higher mobility shift in both JNK isoforms due to covalent binding with the compound. This mobility shift is not seen in cells transfected with MUT JNK plasmids, showing that JNK-IN-8 cannot covalently bind with JNK1 or JNK2 harboring the Cys116Ser mutation. The “Parental” lane contains lysate from un-transfected HEK293T cells and serves as a negative control for the FLAG antibody (Supplementary Figure 1A). Expression of the MUT JNK plasmids also increased c-Jun phosphorylation in cells treated with the JNK-IN-8 and/or lapatinib compared to cells transfected with WT JNK plasmids (Supplementary Figure 1A).

Due to data demonstrating JNK activation by EGFR/HER2 signaling [24, 29] (Figure 1A, 1B), we hypothesized that resistance to lapatinib in TNBCs may be due to elevated JNK activity following lapatinib treatment [36]. Indeed, overexpression of WT JNK1 and JNK2 in the HEK293-T cells significantly increased viability after lapatinib treatment (Supplementary Figure 1B). To determine if JNK-IN-8 sensitizes breast cancer cell lines to lapatinib, cell viability was measured in the presence of either vehicle alone, JNK-IN-8 alone, lapatinib alone, or JNK-IN-8 with lapatinib. Various concentrations of JNK-IN-8 and lapatinib were tested for additive or synergistic effects in TNBC cell lines. Ranges of values tested for JNK-IN-8 were between 0.1μM and 20μM, based on the observed inhibition of c-Jun phosphorylation at 5μM (Figure 1B). Lapatinib concentrations were based on published IC_50_ values for cell lines: 5-10μM for MDA-MB-231 [13, 37] and 3μM for HCC1569 [38]. The IC_50_ for the MDA-MB-436 cell line was presumed to be similar to MDA-MB-231 cells. For Figure 1C, blue circles represent a different combination of JNK-IN-8 and lapatinib concentrations. Combination Indexes (CIs) less than one (the y-axis center) are synergistic, CIs equaling one are additive, and CIs greater than one are antagonistic [39, 40]. For all three cell lines, the combination of JNK-IN-8 and lapatinib synergistically decreased cell viability, especially at combinations causing greater than 50% effect. Cell confluence was measured over time using phase contrast microscopy, also confirming that the JNK-IN-8 and lapatinib combination decreases cell proliferation or increases cytotoxicity greater than vehicle or either compound alone (Supplementary Figure 1C).

A single set of synergistic concentrations that resulted in a ∼70% reduction in viability (effect) was chosen for subsequent apoptosis studies. In Figure 1D, graphs represent percentages of early and late apoptotic cells after 48 hours of treatment for MDA-MB-231 and HCC1569 cells, and 72 hours of treatment for MDA-MB-436 cells. For all cell lines, apoptosis was significantly higher with combination treatment than with control or either compound alone, indicating that the synergistic effect is due, at least in part, to apoptosis. Bright field pictures of MDA-MB-231 cells after 72 hours of treatment confirm the presence of rounded, apoptotic cells exhibiting membrane blebs (Supplementary Figure 1D).

To test the *in vivo* efficacy of combining JNK-IN-8 and lapatinib, nude female mice were injected orthotopically with human MDA-MB-231 cells. Once tumors reached an average volume of 80mm^3^, treatment with vehicle, JNK-IN-8 (25mg/kg), lapatinib (75mg/kg), or JNK-IN-8 and lapatinib combination began. Tumor growth was assessed using a Kaplan-Meier curve measuring time to the attainment of maximal tumor size (Figure 1E). Median time to maximum tumor growth for each group occurred at 15 days (Vehicle), 15 days (JIN), 12 days (Lap), and 21.5 days (J+L). Using a Log Rank (Mantel Cox) test, the vehicle, lapatinib, and JNK-IN-8 treatment curves were all found to be shorter than the combination of JNK-IN-8 and lapatinib (p=0.0248, p=0.0014 and p=0.0507, respectively). Vehicle, lapatinib, and JNK-IN-8 alone were all significantly inferior to the combination treatment using the Gehan-Breslow-Wilcoxon Test (p=0.0290, p=0.0019, and p=0.0190, respectively), implying greater synergy during earlier stages of tumor growth. The tumor from one mouse in the combination group did not reach maximum size by the end of the experiment at Day 30.

### JNK-IN-8 and lapatinib synergize largely through targeting JNK1 and HER2, respectively

**Figure 2:**
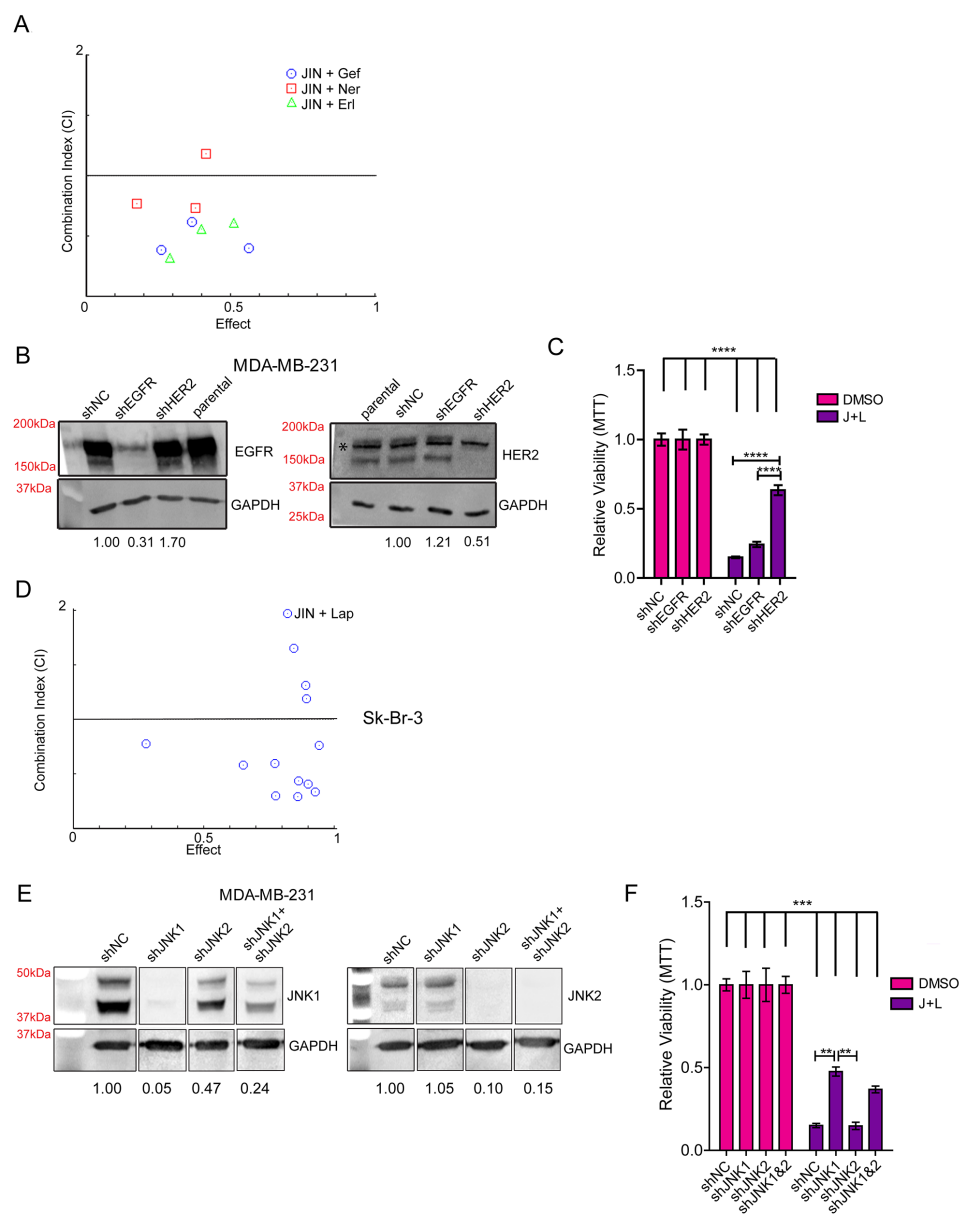
HER2 and JNK1 are Necessary for Maximum Synergy between JNK-IN-8 and Lapatinib **(A)** MDA-MB-231 cells were treated with 5μM JNK-IN-8 (JIN) and/or 1, 5, and 10μM gefitinib (Gef), 0.1, 0.5, and 1.0μM neratinib (Ner), or 1, 5, and 10μM erlotinib (Erl). MTT values were used to calculate synergy using CompuSyn software. Combination Indexes (CIs) are plotted against Effect (% decreased cell viability by MTT). Points below 1.0 CI (median line) are synergistic. **(B)** MDA-MB-231 cells stably expressing either shNC (non-silencing control), shEGFR, or shHER2 and their relative densitometries to shNC for EGFR and HER2 expression by western blot. The non-specific band (denoted by the asterisk “_*_”) in the HER2 blot was included in densitometry calculation due to its close proximity to HER2. **(C)** Cell lines with indicated shRNA plasmids were treated with vehicle (DMSO) or 3μM lapatinib and 5μM JNK-IN-8 (J+L) for 72 hours. Cell viability was assayed using MTT and bars represent relative viability compared to vehicle. **(D)** Sk-Br-3 cells were treated with various concentrations of J+L for 72hrs and assayed for viability using MTT. MTT values were used to calculate synergy using CompuSyn software. Combination Indexes (CIs) are plotted against Effect (% decreased cell viability by MTT). Points below 1.0 CI (median line) are synergistic. **(E)** MDA-MD-231 cells stably expressing either shNC, shJNK1, shJNK2, or shJNK1 and shJNK2 were lysed for western blot. Their relative densitometries to shNC for JNK1 and JNK2 expression are shown. **(F)** Cell lines with indicated shRNA plasmids were treated with DMSO or J+L for 72 hours. Cell viability was assayed using MTT and bars represent relative viability compared to vehicle.

To interrogate the specificity of lapatinib in TNBC cells, we first tested for synergy between JNK-IN-8 and other EGFR or EGFR/HER2 inhibitors. MDA-MB-231 cells were treated with three concentrations of the reversible EGFR inhibitors gefitinib and erlotinib based on published IC_50_ values for breast cancer cell lines [41–44] in combination with 5μM JNK-IN-8. The EGFR/HER2 covalent inhibitor neratinib [45] was also used. For almost all of the concentrations tested, JNK-IN-8 was synergistic with these compounds in decreasing cell viability as shown by CIs less than one (Figure 2A). The ability of JNK-IN-8 to synergize with each of these inhibitors supports the notion that EGFR and HER2 are critical targets of this response, and that lapatinib's synergy with JNK-IN-8 is not due to an off-target effect.

The “triple-negative” designation of TNBC refers to a lack of HER2 overexpression, not a complete lack of expression [46], as our laboratory (Figure 2B) and others [13, 47, 48] have shown. To decipher whether EGFR or HER2 is more important for synergy between JNK-IN-8 and lapatinib, we stably knocked down EGFR or HER2 expression in MDA-MB-231 cells (Figure 2B). Compound knockdown of both receptors could not be achieved due to lethality caused by the loss of both receptors. These cells were treated with vehicle or the combination of 3μM lapatinib and 5μM JNK-IN-8 for 72 hours in full media. Despite frequently elevated EGFR expression in TNBCs, Figure 2C suggests that inhibition of EGFR is not necessary for significant synergy between JNK-IN-8 and lapatinib, as its knockdown does not rescue cells from combination treatment-induced cell death. In contrast, shHER2 cells were significantly more resistant to the JNK-IN-8 and lapatinib combination, supporting the notion that HER2 is a critical target of lapatinib for synergy with JNK-IN-8 (Figure 2C). The lack of total viability rescue could indicate some level of off-target effects, which occur frequently with pharmacologic inhibitors of kinases, and may occur more easily when the preferred targets of the inhibitors are in low abundance. However, Figure 2A would support the notion that lapatinib is acting specifically, and lack of total rescue is likely due to residual HER2 expression.

To explore the unexpected conclusion that HER2 is the key target of lapatinib for its synergy with JNK-IN-8, we tested whether the combination also synergizes in HER2-amplified cells. Using the Sk-Br-3 cell line [49], we observed that JNK-IN-8 and lapatinib synergistically inhibited cell viability (Figure 2D). These data agree with the conclusion that HER2 inhibition by lapatinib is more important for JNK-IN-8 and lapatinib synergy than EGFR inhibition.

We then used a similar approach to evaluate the importance of JNK1 or JNK2 inhibition in sensitizing TNBC cells to lapatinib. Using lentiviral transduction to stably knockdown expression, we obtained 95% and 90% inhibition of JNK1 and JNK2 expression, respectively. Compound JNK1 and JNK2 knockdown was less efficient. The highest achievable double knockdown was approximately 76% of JNK1 with 85% of JNK2 (Figure 2E). JNK1 knockdown, and to a lesser extent compound JNK1 and JNK2 knockdown, significantly rescued cell viability when treated with JNK-IN-8 and lapatinib combination. Knockdown of JNK2 alone had no effect on synergy (Figure 2F). This indicates that inhibition of JNK1 is necessary for full synergy between JNK-IN-8 and lapatinib, and that the level of JNK1 knockdown directly correlates with the level of rescue. Full rescue was not observed in any of the JNK knockdown experiments, suggesting that even very low JNK expression/activity enhances the effect of JNK-IN-8 and lapatinib.

The result that JNK1 inhibition by JNK-IN-8 is important for synergy with lapatinib is not surprising considering that JNK-IN-8 was shown to have greater activity toward purified, recombinant JNK1 *in vitro* [34]. The activity of JNK-IN-8 was explored in cell lines produced from spontaneous mammary tumors in JNK1−/− or JNK2−/− mice [50] in order to determine whether JNK-IN-8 has greater activity toward JNK1 within a cellular context. We determined that inhibition of JNK1 and JNK2 by JNK-IN-8 was approximately equivalent in regards to decreased phosphorylation of c-Jun at the JNK specific, serine 63 site. However, phosphorylation of JNK2 was inhibited by about 80% after JNK-IN-8 treatment, while phospho-JNK1 was only inhibited by about 20% (Supplementary Figure 2A). This indicates that the inhibition of endogenous JNK1 by JNK-IN-8 is not greater than inhibition of JNK2. Overall, these results lead us to conclude that the effect seen after JNK1 knockdown in Figure 2F is not due to a stronger effect on JNK1 by JNK-IN-8, but rather highlights the specific importance of JNK1 in the context of lapatinib treatment.

### Lapatinib treatment activates JNK, but decreases AP-1 activity

**Figure 3:**
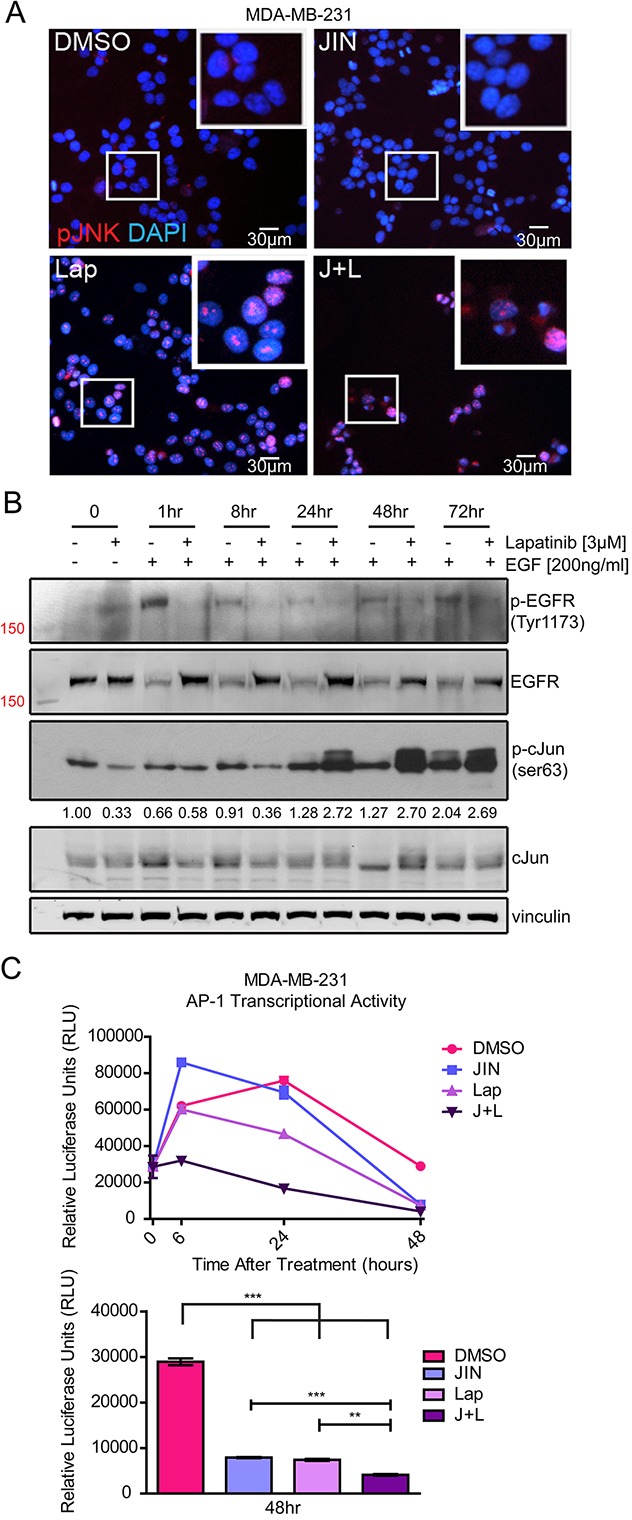
Lapatinib Affects JNK and AP-1 Signaling **(A)** Immunofluorescence of MDA-MB-231 cells treated with vehicle (DMSO), 5μM JNK-IN-8 (JIN), and/or 3μM lapatinib (Lap) for 72 hours. Cells were stained with phospho-JNK (Thr183/Tyr185)(red) and nuclei were counterstained with DAPI (blue). **(B)** MDA-MB-231 cells were serum-starved overnight and pre-treated with 3μM lapatinib or DMSO for 3 hours. After that time, cells were lysed (0hr) or stimulated with EGF for 1, 8, 24, 48, and 72 hours. Western blots of phospho-EGFR (Tyr1173), EGFR, phospho-cJun (ser63), cJun, and vinculin are shown with band densitometries beneath. **(C)** MDA-MB-231 cells were transfected with an AP-1 luciferase reporter plasmid. Cells were treated for the indicated time points with vehicle (DMSO), 5μM JIN and/or 3μM Lap. The bar graph represents AP-1 transcriptional activity at 48 hours.

Although EGF activates JNK activity as in Figure 1A, lapatinib increased nuclear localization of phosphorylated JNK after 72 hours of treatment in full media (Figure 3A, “Lap” inset). These data suggest that intrinsic lapatinib resistance of TNBC cell lines involves JNK activation during lapatinib exposure.

Lapatinib, in line with increasing JNK phosphorylation (Figure 3A), increases nuclear c-Jun (Supplementary Figure 3A). These events were observed in full serum. Experiments using EGF in serum free media showed a similar result where lapatinib inhibited phospho-c-Jun (S63) at early time points (Figure 3B), consistent with JNK being downstream of EGFR/HER2 signaling, however, at later time points c-Jun phosphorylation was increased similarly to phospho-JNK in Figure 3A. Compensatory signaling in breast cancer cells is frequently attributed to resistance mechanisms to kinase inhibitors including lapatinib [14, 51–53]. Together, these data imply that other factors compensate for lost JNK and c-Jun activation after exposure to lapatinib, consistent with our hypothesis that JNK signaling may confer resistance to lapatinib.

AP-1 activity, which is regulated by c-Jun and various other transcription factors [54–56] was evaluated as a potential downstream response to lapatinib and JNK-IN-8 treatment. In the MDA-MB-231 cells, AP-1-driven luciferase activity decreased significantly after lapatinib treatment, however, treatment with both compounds maximally inhibited AP-1 activity even at early time points (Figure 3C). These results implicate the regulation of AP-1 transcription factors other than c-Jun by EGFR/HER2, such as fos family members [57–59], whose inhibition leads to an overall decrease in AP-1 activity.

### JNK-IN-8 and lapatinib cause accumulation of cytotoxic oxidative stress

**Figure 4:**
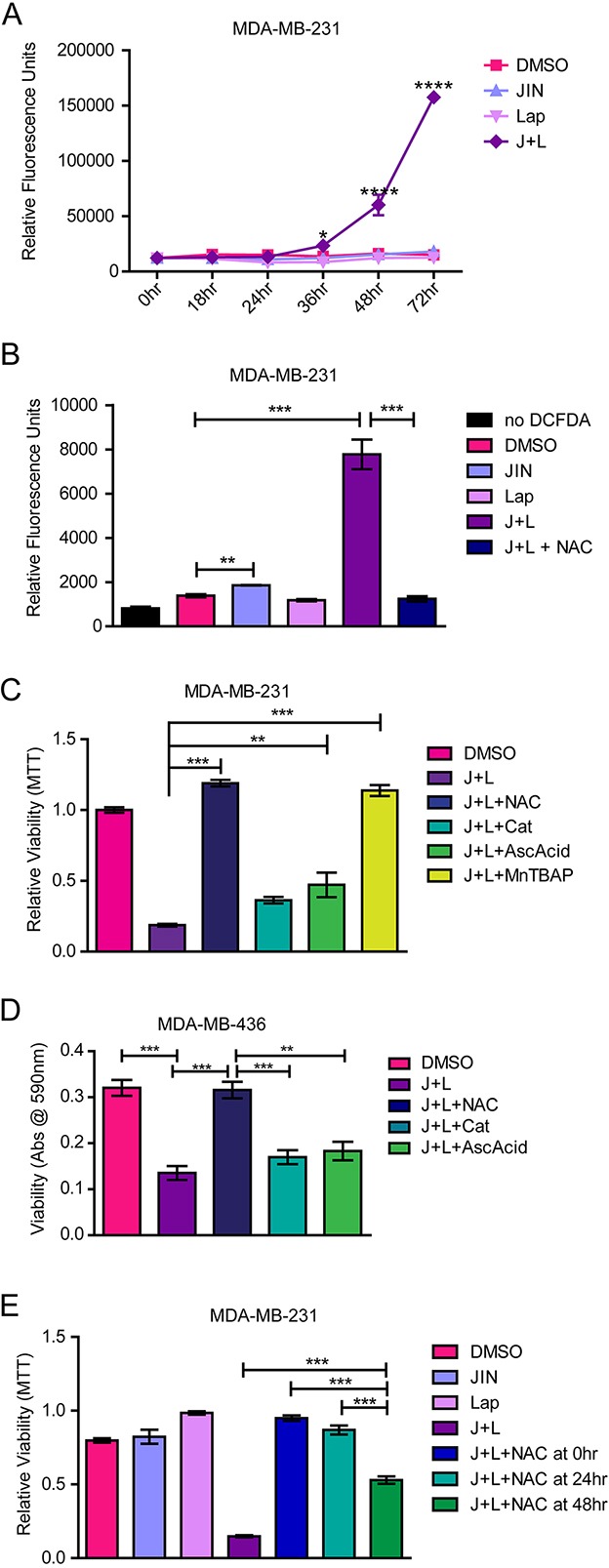
JNK-IN-8 and Lapatinib Cause Cell Death by Increasing Reactive Oxygen Species **(A)** MDA-MB-231 cells were treated with either vehicle (DMSO), 3μM JNK-IN-8 (JIN) and/or 5μM lapatinib (Lap). ROS levels were assayed using 2′,7′ –dichlorofluorescein diacetate (DCDFA). Increased fluorescence indicates increased ROS. **(B)** Bar graph representing amount of ROS by DCDFA at 72 hours after treatment. 5mM NAC was used for ROS quenching. **(C)** MDA-MB-231 and **(D)** MDA-MB-436 cells were treated with DMSO, 3μM JIN and/or 5μM Lap, or JNK-IN-8 and lapatinib (J+L) with 5mM NAC, 500 U/mL catalase(Cat), 100μg/mL ascorbic acid (AscAcid), or 100μM MnTBAP for 72 hours in full media. Bar graphs represent viability by MTT assay. **(E)** NAC was either added at the same time as J+L (NAC at 0hr), 24 hours after J+L (NAC at 24hr), or 48 hours and after J+L (NAC at 48hr). Cell viability at 72 hours after addition of J+L was assayed using MTT.

Cells sensitive to EGFR/HER2 inhibition exhibit increased oxidative stress after lapatinib treatment [18], so we examined whether JNK inhibition might restore this mechanism of cell death. Indeed, elevated ROS were noted beginning at 36 hours and exhibited a 10-fold increase by 72 hours after treatment with the JNK-IN-8 and lapatinib combination (Figure 4A). Notably, neither compound alone increased ROS levels. This finding indicates that either EGFR/HER2 or JNK sustains an oxidative balance while the other is inhibited.

To determine whether elevated ROS is merely a consequence of apoptosis, cells were treated with JNK-IN-8 and lapatinib in addition to various ROS scavenging molecules: N-acetyl cysteine (NAC), ascorbic acid, catalase, and Manganese (III) tetrakis (4-benzoic acid) porphyrin (MnTBAP) (a Super Oxide Dismutase (SOD) mimetic). These compounds represent major cellular defenses against oxidative stress: glutathione, SOD1/2 and peroxyredoxin [60]. Treatment of MDA-MB-231 cells with NAC, the precursor for glutathione, for 72 hours in the presence of JNK-IN-8 and lapatinib completely abrogated ROS accumulation (Figure 4B), and rescued cell viability (Figure 4C, 4D). Ascorbic acid also significantly rescued cell viability, although not nearly to the degree that NAC did, whereas catalase was ineffective. MnTBAP also rescued viability in the presence of JNK-IN-8 and lapatinib (Figure 4C). Similar results were achieved with the MDA-MD-436 cell line (Figure 4D). These results indicate that elevated oxidative stress after JNK-IN-8 and lapatinib treatment causes apoptosis.

The timing of NAC addition was important to the degree of protection in the MDA-MB-231 cells. NAC added at the same time as JNK-IN-8 and lapatinib resulted in complete protection, whereas addition of NAC after JNK-IN-8 and lapatinib resulted in diminishing rescue (Figure 4E). Noticeably, there was no significant change from untreated controls when cells were treated with NAC 24 hours after JNK-IN-8 and lapatinib, but about a 50% drop-off in rescue resulted when cells were treated with NAC 48 hours after JNK-IN-8 and lapatinib. Nevertheless, JNK-IN-8 and lapatinib combination rescue was still significant at this time point (Figure 4E). This corresponds with the ROS time course showing that ROS become elevated between 24 and 48 hours after JNK-IN-8 and lapatinib treatment in the MDA-MB-231 cells. To show that ROS scavengers are not simply sequestering JNK-IN-8 or lapatinib in the medium (or vice versa), we repeated viability assays with the compounds washed out prior to NAC treatment. Under these conditions, NAC significantly rescued the viability of MDA-MB-231 and MDA-MB-436 cells pre-treated with JNK-IN-8 and lapatinib compared to full media alone (Supplementary Figure 4A). These data support that NAC rescues cells from oxidative cellular injury and death imposed by lapatinib and JNK-IN-8.

### Elevated oxidative stress is associated with a decreased antioxidant response

NFκB can promote an antioxidant response through regulation of genes such as Ferritin Heavy Chain (FHC), Mn-SOD (SOD2), and SOD1 [61–65], as well as genes that are necessary for glutathione biosynthesis [66]. The transcription factor Nrf2 binds the Antioxidant Response Element (ARE) to increase transcription of genes necessary for the production and regeneration of thioredoxin, glutathione, peroxiredoxin, and NADPH [67–72]. Together with AP-1, these transcription factors are major regulators of the antioxidant response with each controlling targets that eliminate cytotoxic ROS from the cell [60].

**Figure 5:**
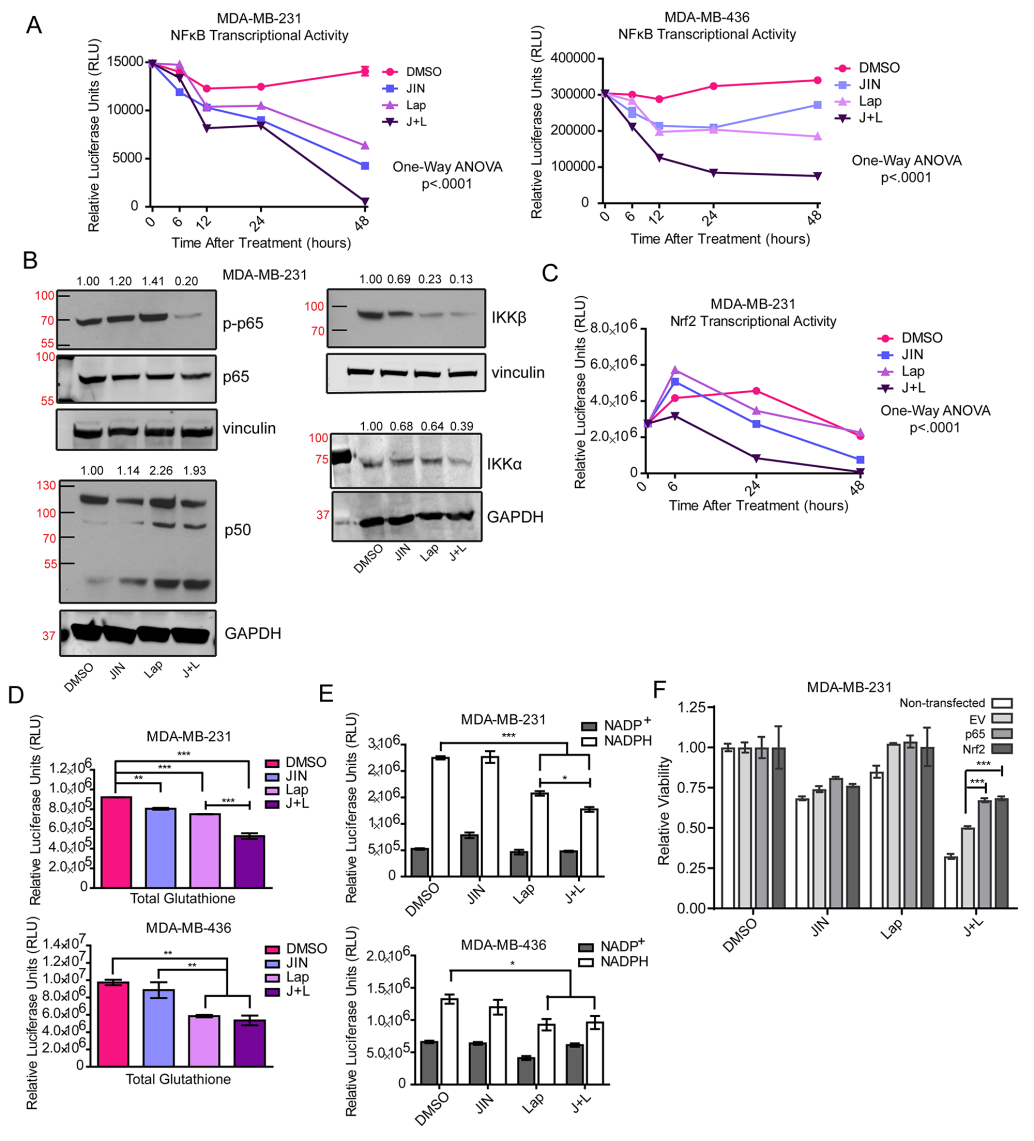
JNK-IN-8 and Lapatinib Decrease Important Antioxidants **(A)** Cells were treated with 5μM JNK-IN-8 (JIN) and/or 3μM lapatinib (Lap) for MDA-MB-231 cells, and 4μM JIN and/or 7μM Lap for MDA-MB-436 cells for various time points. Cells were also transfected with NFκB luciferase reporter plasmids. Line graph points represent normalized luciferase values from 3 technical replicates. **(B)** MDA-MB-231 cells were treated with either vehicle (DMSO), 5μM JIN and/or 3μM Lap for 48 hours in full media. Whole cell lysates were probed for phospho-p65(ser536), p65, p50, IKKβ, and IKKα. **(C)** MDA-MB-231 cells were treated with 5μM JIN and/or 3μM Lap for various time points and transfected with a luciferase reporter plasmid under the control of the NQO1 promoter containing Nrf2/ antioxidant response elements (ARE). Line graph points represent normalized luciferase values from 3 technical replicates. **(D)** Relative levels of glutathione were measured in MDA-MB-231 and MDA-MB-436 cells after 72 hours of treatment in DMSO, JIN, Lap, or JNK-IN-8 and lapatinib (J+L). **(E)** Levels of NADP^+^ and NADPH were measured in MDA-MB-231 and MDA-MB-436 cells after 72 hours of treatment in DMSO, JIN, Lap, or J+L. **(F)** MDA-MB-231 cells were non-transfected, transfected with empty vector (EV), or expression plasmids for p65 (p65 OE) and Nrf2 (Nrf2 OE). Twenty four hours after transfection cells were treated with either vehicle (DMSO), 5μM JIN and/or 3μM Lap for 72 hours in full media. Cell viability was assayed using MTS.

From 12 to 48 hours of treatment, JNK-IN-8, lapatinib, and the combination all significantly inhibit NFκB transcriptional activity in MDA-MB-231 cells. Values are less than half that of vehicle-treated cells for JNK-IN-8 and lapatinib alone, and negligible for the combination treatment (Figure 5A). In MDA-MB-436 cells, JNK-IN-8 and lapatinib have less effect alone, but NFκB transcriptional activity is again strongly inhibited by the combination treatment (Figure 5A). By western blot analysis, treatment with JNK-IN-8 and lapatinib did not significantly decrease the expression of p65 or p50 subunits of the canonical NFκB pathway (Figure 5B). However, phosphorylation of p65 at serine 536, an activating phospho site of p65, was significantly decreased by the combination treatment. In addition, Inhibitor of kappa B (IκB) Kinase (IKK) α and β expression were consistently decreased with combination treatment (Figure 5B) suggesting that decreased IκB dissociation from NFκB in addition to a decrease in activating phosphorylation may be lowering NFκB transcriptional activity.

We also investigated the transcriptional activity at the NAD(P)H Dehydrogenase Quinone 1 (NQO1) promoter (containing AREs) which is positively regulated by the Nrf2 transcription factor [71, 72]. Similar to NFκB, significant decreases are seen in as little as 6 hours with JNK-IN-8 and lapatinib combination in MDA-MB-231 cells. By 48 hours, both JNK-IN-8 alone and JNK-IN-8 and lapatinib combination significantly decreased Nrf2 transcriptional activity through the ARE (Figure 5C).

To determine what biological effects reduced NFκB and Nrf2 signaling may have on the antioxidant response, we quantified relative amounts of glutathione (Figure 5D) and NADP^+^/NADPH (Figure 5E) using luciferase-based assays, and quantified thioredoxin and Heme Oxygenase-1 (HO-1) levels by western blot (Supplementary Figure 5A, 5B). Total levels of glutathione decreased significantly in JNK-IN-8 and lapatinib combination-treated cells compared to vehicle in MDA-MB-231 cells. In MDA-MB-436 cells, both lapatinib alone and JNK-IN-8 and lapatinib combination significantly reduce glutathione. NADPH, necessary for the reduction of glutathione and thioredoxin [73], also declined in a similar pattern (Figure 5E). Total levels of thioredoxin remained unchanged by treatment (Supplementary Figure 5A), and HO-1 (Supplementary Figure 5B) differences were not consistent. This indicates that expression of some Nrf2 targets persists, however not enough to control accumulation of ROS.

The necessity of NFκB and Nrf2 expression in controlling oxidative stress after JNK-IN-8 and lapatinib combination is evident in Figure 5F. Overexpression of p65 or Nrf2 (Supplementary Figure 5C) each significantly rescue cell viability of MDA-MB-231 cells being treated with JNK-IN-8 and lapatinib combination compared to cells expressing an empty plasmid (Figure 5F). Data are shown relative to each group's vehicle treatment, revealing that transfected cells do not respond as well to the combination treatment. This is likely due to overall lower viability in transfected cells compared to untransfected cells (data not shown). While the rescue is significant, it does not reach the level of viability of cells treated with vehicle, possibly indicating the need for rescue of NFκB and Nrf2 in combination to fully combat the effects of JNK-IN-8 and lapatinib.

Together, these data support that lapatinib and JNK-IN-8 inhibit signaling pathways integral for cancer cell survival during oxidative stress, namely NFκB and Nrf2, leading to glutathione depletion. Consistent with this finding is that repletion of glutathione, using NAC supplementation, rescues cell viability. In addition, decreased levels of NADPH would inhibit the reduction of oxidized glutathione and thioredoxin, preventing the recycling of these important antioxidants.

## DISCUSSION

Neither EGFR nor EGFR/HER2 inhibitors alone show efficacy in TNBC despite relatively high EGFR expression. Presented in this work, we established a possible therapeutic benefit for the combination of lapatinib and JNK-IN-8 in the treatment of TNBC. JNK-IN-8 and lapatinib synergize to cause apoptosis in three cell line models of TNBC: MDA-MB-231 (expressing mutant BRAF and KRAS), MDA-MB-436 (expressing mutant BRCA-1 and Rb-1), and HCC1569 (expressing mutant PTEN) [48, 74–76], as well as the HER2-amplified SK-Br-3 cells [77]. Cell line specific findings are summarized in Supplementary Table 1. These are the first studies to show that dual blockade of JNK and EGFR/HER2, through JNK-IN-8 and lapatinib treatment, cooperate to synergistically decrease cell viability by elevating oxidative stress.

**Figure 6:**
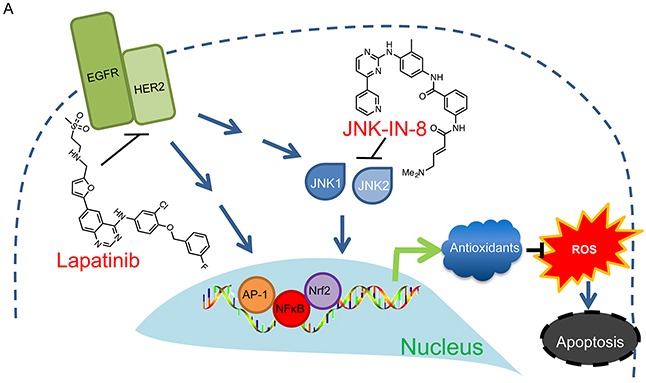
Proposed Mechanism of JNK-IN-8 and Lapatinib Synergy Lapatinib and JNK-IN-8 jointly inhibit the transcriptional activities of AP-1, NFκB, and Nrf2. This disrupts the cell's natural antioxidant response resulting in accumulation of ROS leading to apoptosis.

Cancer cells tolerate high levels of ROS by upregulating antioxidants [78–80]. Elevated NFκB, AP-1, and Nrf2 in cancer cells contribute to their antioxidant response [60, 72, 81]. Their targets that participate in the antioxidant response include sulfiredoxin, genes for glutathione and thioredoxin biosynthesis, and Mn-SOD [60, 66, 81–83]. In addition to its antioxidant targets, AP-1 can enhance the transcriptional activity of NFκB through various mechanisms [84–87], and it can enhance Nrf2 activity as AP-1 consensus sequences often lie within AREs [82]. Therefore, decreased AP-1, NFκB, and Nrf2 transcriptional activities following JNK-IN-8 and lapatinib combination treatment may jointly lower cellular antioxidant response. While it was noted that JNK-IN-8 and lapatinib alone were capable of inhibiting multiple targets, it is important to observe that some targets are decreased more by JNK-IN-8 than lapatinib and vice versa. This led us to conclude that it is the combination of these changes that causes an overall decrease in antioxidant response and apoptosis. This mechanism is strongly supported by Figure 5A showing that only the combination of JNK-IN-8 and lapatinib increased overall oxidative stress while the single compounds had no effect. This indicates that signaling through EGFR/HER2 and JNK compensate for one another in the induction of proper antioxidant responses, highlighting the necessity for the combination of both compounds over single agent treatment. The proposed mechanism is summarized in Figure 6.

With regard to further improving the *in vivo* efficacy of this combination, the doses of JNK-IN-8 and lapatinib used in the tumor xenograft experiment were very well tolerated and could be increased. Further pharmacokinetic and pharmacodynamic studies of JNK-IN-8 will be needed to assess the ideal dose, vehicle, and administration route. However, we are encouraged by the data observed where the combination of JNK-IN-8 and lapatinib significantly slows tumor growth in mice compared to those treated with vehicle or single agents alone.

Consistent with our hypothesis that JNK conveys intrinsic or acquired resistance to lapatinib, JNK-IN-8 was recently used to overcome resistance to a BRAF inhibitor in melanoma cells [36]. This highlights the importance of JNK as a regulator of multiple signaling pathways, and its attractiveness as a “druggable” target in cancer. These studies are the first to report the benefit of inhibiting JNK in combination with lapatinib. With further testing, this combination may be a viable option to treat patients with aggressive TNBC, whose lack of molecularly-targeted therapies represents an urgent and unmet medical need. Furthermore, testing of this combination in tumor types that are more sensitive to oxidative stress should reveal an even greater clinical impact, and JNK-IN-8 may synergize with other growth factor receptor-targeted therapeutics.

## MATERIALS AND METHODS

### Cell lines and reagents

MDA-MB-436 and HCC1569 cell lines were a kind gift from the lab of Dr. Mark Pegram at Stanford University. They were maintained in RPMI media (# SH30027.01 HyClone, Logan, UT) supplemented with 10% Fetal Bovine Serum (FBS) (#100-106, Gemini Bio Products, Sacramento, CA) at 37°C in a 5% CO_2_ atmosphere. The MDA-MB-231 cell line was purchased from ATCC (Manassas, VA) and maintained in Improved MEM (#A10488-01, Gibco, Grand Island, NY) supplemented with 10% FBS and 10μg/mL Humulin insulin (#HI-310, Eli Lilly & Co., Indianapolis, IN). HEK-293-T cells were obtained from ATCC (Manassas, VA) and were maintained in DMEM with high glucose (#SH30243.01 HyClone, Logan, UT) supplemented with 10% FBS at 37°C in 5% CO_2_. JNK-IN-8 (synthesized by Dr. Ramakrishna Edupuganti from the Targeted Therapeutic Drug Discovery and Development Program at the University of Texas according to [21]) and lapatinib (#L-4804 LC Labs, Woburn, MA) were dissolved in dimethyl sulfoxide (DMSO). Gefitinib was purchased from #H018 AK Scientific, Union City, CA, Erlotinib from #10483 Cayman Chemical, Ann Arbor, MI, and Neratinib from #52150 SelleckChem, Houston, TX. Epidermal Growth Factor (EGF) was purchased from PeproTech (#100-15 Rocky Hill, NJ). ROS detection reagent DCDFA (2′,7′ –dichlorofluorescein diacetate) was purchased from Abcam (#ab113851, Cambridge, MA). N-acetyl cysteine (NAC)(#A9165), Ascorbic Acid(#A5960), and Catalase(#C-40) were purchased from Sigma-Aldrich (St. Louis, MO).

### Western blot

Phospho-c-Jun(Ser63)(#9261), phospho-JNK (Thr 183/Tyr185)(#9251), total c-Jun (#9165), EGFR (#4267), pEGFR (#4407), IKKβ(#2684), p65(Ser536)(#3033), p65 (#8242), JNK1 (#3708), JNK2 (#9258), and HO-1 (#5053) were purchased from Cell Signaling (Danvers, MA). Primary Cell Signaling antibodies were used at a 1:1000 dilution, phospho- antibodies were used at 1:500. TRX (#271281)(1:1000), HER2 (#284)(1:600 dilution), IKKα (#7606)(1:250 dilution) were purchased from Santa Cruz Biotechnology (Dallas, TX). FLAG epitope antibody (F3165) was purchased from Sigma-Aldrich (St. Louis, MO). GAPDH (#6C5) antibody was purchased from Advanced Immunochemical (Long Beach, CA) and used at a dilution of 1:2500 for 20 minutes at room temperature. Actin antibody (MAB1501) was purchased from EMD Millipore (Darmstadt, Germany). Cells were lysed in Radio Immunoprecipitation Buffer (RIPA) (50 mM Tris-HCl (pH 7.4), 1% NP-40, 0.25% Na-deoxycholate, 150mM NaCl, 1mM EDTA) and 80μg of protein was separated using SDS-PAGE. 100μg of protein was loaded for HER2 blots and 40μg of protein was loaded for EGFR blots.

Proteins were then transferred to 0.22μm nitrocellulose and blocked in 5% non-fat powdered milk in TBS-T (Tris base, NaCl, pH 7.6 plus 0.05% Tween 20) for one hour, shaking at room temperature. Nitrocellulose blots were incubated overnight at 4°C on a rocking platform in the presence of primary antibody. After removal of primary antibody by washing in TBS-T, blots of primary antibodies made in mouse were incubated with HRP-conjugated anti-mouse IgG secondary antibody (#2005 Santa Cruz Biotechnology, Dallas, TX) at a 1:1000 dilution. Blots of primary antibodies made in rabbit were incubated in anti-rabbit IgG secondary antibody (#7074 Cell Signaling, Danvers, MA) at a 1:1000 dilution.

Proteins were visualized using ECL 2 (#80196 Thermo Scientific Grand Island, NY) to produce fluorescent signal that was detected by scanning with the Storm 860 Imager (GE Amersham Pittsburgh, PA). Minimal, linear adjustment of upper and lower input bounds were accomplished using Photoshop V7 SP1 (Adobe, San Jose, CA). Band densitometries were calculated using Image J software (National Institutes of Health Bethesda, MD). Molecular weight ladder (#1610374, Biorad) bands are designated on the figures where possible.

### Cellular viability assay using MTT or MTS

Cells were plated at 40% confluency in 96-well, flat bottom plates. After being allowed to attach overnight, cells were treated with vehicle JNK-IN-8, lapatinib, or JNK-IN-8 and lapatinib combination for various timepoints. After treatment, MTT (3-(4,5-Dimethylthiazol-2-yl)-2,5-diphenyltetrazolium bromide) (#475989 Calbiochem, San Diego, CA) was added to a final concentration of 0.5mg/ml from 5mg/ml stock in PBS. Cellular metabolism reduces this tetrazole to insoluble formazan crystals. After four hours of incubation, the media + MTT was gently removed. Formazan crystals were dissolved using DMSO and absorbance at 590nm was read using the Synergy II Plate Reader (BioTek Winooski, VT). For viability assays using MTS, the Promega (Madison, WI) CellTiter 96® AQ_ueous_ One Solution was used according to manufacturer's instructions. Absorbance values were read at 490nm using the Victor^3^V Model 1420 plate reader from PerkinElmer (Waltham, MA).

### Cell proliferation assay using phase contrast microscopy

Two thousand cells were plated in 96-well plates and allowed to attach overnight. The next day compounds were added in full media and plates were placed immediately into the Incucyte® ZOOM™ live cell imaging system from Essen Bioscience (Ann Arbor, MI). Cell confluence was imaged every hour for 60 hours and data was analyzed with Incucyte software using the basic confluency algorithm.

### Synergy calculation

Raw absorbance values from MTT assays were normalized to their respective vehicle control wells and represented as a % decrease in absorbance (control = 0 effect, 2% decrease in absorbance =.02 effect). These values were entered into the CompuSyn software (ComboSyn, Paramus, NJ) as “effect” along with the concentrations of both JNK-IN-8 and lapatinib used to obtain that value. Effect values for increasing concentrations of each drug alone were used to calculate the median-effect, which was then used to determine whether the effect caused by a combination concentration was synergistic [25]. Combination Index plots and values were generated by the CompuSyn software.

### Annexin V apoptosis assay

Apoptosis was measured by Annexin V and propidium iodide (PI) positivity using the “Rapid-binding protocol” from the Calbiochem Annexin V-FITC kit (#PF032 San Diego, CA). Fluorescence from the Annexin V antibody or PI was read using the Guava EasyCyte™ flow cytometer (Millipore, Darmstadt, Germany). Analysis and generation of two-color plots were performed using the FlowJo 10 software (Ashland, OR).

### Statistics

All bar graph statistical analyses were performed using Prism5 software (GraphPad LaJolla, CA). One-way or Two-way ANOVA was performed followed by Tukey's post-test. For all graphs: (^*^≤.05, ^**^≤.01, ^***^≤.001, ^****^≤.0001). Unless otherwise noted, graphs are created from the data of three biological replicates. Other statistical analyses are mentioned in the text or figure legends.

### Xenograft mouse tumors

Nude female mice (nu/J #002019, Jackson Labs, Sacramento, CA) at 6 weeks of age were injected orthotopically (L4 mammary gland) with 3×10^6^ MDA-MB-231 TNBC cells in PBS. Nine days later, tumors reached an average volume of 80mm^3^ and mice were randomized into four groups (n=10 mice per group). Treatment with vehicle, JNK-IN-8 (25mg/kg) (Advanced ChemBlocks, Burlingame, CA), lapatinib (75mg/kg) (MedChem Express, Monmouth Junction, NJ), or JNK-IN-8 and lapatinib combination was administered daily. JNK-IN-8 or its vehicle (2% ethanol and 5% Tween-80 in PBS) were administered by intraperitoneal injection, and lapatinib or its vehicle (0.5% hydroxypropylmethylcellulose and 0.1% Tween-80 in PBS) were administered by oral gavage. Tumors were measured every other day and mice were monitored for signs of distress including significant weight loss, loss of ambulation, or difficulty breathing. Mice with tumors that reached a maximum diameter of 15mm or a maximum volume of 750mm^3^ were euthanized. A Kaplan-Meier curve is shown where an adverse event represents attainment of the maximal tumor size. No mice died unexpectedly during this experiment and were treated humanely with oversight by the University of Texas IACUC committee (protocol# AUP-2015-00170).

### Light microscopy

An Olympus CKX41 (Center Valley, PA) upright light microscope with a phase-contrast filter was used to visualize cells in culture. Brightfield photographs of cells were taken at 1000X using QCapturePro imaging software (QImaging, Surrey, BC, Canada).

### Target gene knockdown with retroviral shRNA

HEK-293-T cells were plated at 1×10^6^ cells per 60mm dish on poly-lysine coated plates and allowed to attach overnight. The next day, six total μg of DNA was mixed with 6uL of Enhancer™ Reagent and complexed with 6uL of Lipofectamine® 3000 (#L3000001 Life Technologies, Grand Island, NY) per the manufacturer's protocol. For the DNA mixture, 3μg of shRNA plasmid and 3μg of pCL-Eco (Open Biosystems Lafayette, CO) was used. This mixture was allowed to sit for 10 minutes and added drop-wise to HEK-293-T cells. Target MDA-MB-231 cells were plated the day after transfection at 250,000 cells per 60mm dish. At 48 hours and 72 hours after transfection, media containing retrovirus was taken off the HEK-293-T cells and filtered through a 0.45μM syringe filter to remove HEK-293-T packaging cells. Polybrene (Hexadimethrine bromide) (#107689 Sigma, St. Louis, MO) at 8ug/mL was added to increase viral transduction efficiency and this mixture was added drop-wise to the MDA-MB-231 target cells. After 72 hours of incubation in viral media, fresh media containing G418 (#29065A Santa Cruz Biotechnology Dallas, TX) at 800μg/mL or puromycin at 2μg/mL was added to select for cells expressing the shRNA constructs. ShRNA retroviral plasmids: shEGFR (targeting sequence: CTGTGCAGAATCCTGTCTATC), shHER2 (targeting sequence: GGGAGAGAGTTCTGAGGATTG) and shNC (control) (targeting sequence: CAACAAGATGAAGAGCACCAA) sequences in the pSUPERIOR.retro.neo.gipz vector were obtained from Dr. Dennis Hughes at the MD Anderson Cancer Center (Houston, TX). shJNK1 (NM_002750) and shJNK2 (NM_139068) sequences in the pGIPZ-lenti-puro plasmid were obtained from Open Biosystems (Lafayette, CO).

### Immunofluorescence

Cells were plated at 25% confluency in 8-well chamber slides and treated for 48 and/or 72 hours. Media was removed, cells were washed twice with cold PBS and fixed in ice-cold methanol:acetone 1:1 for 10 minutes at −20°C. Fixative was removed and cells were washed twice more with cold PBS before incubation for 12 minutes on ice in permeabilization buffer (0.05% TritonX-100 in PBS). Cells were blocked in 5% normal goat serum in PBS for one hour at room temperature. Phospho-JNK(Thr183/Tyr185) (#9251) and c-Jun (#9165) primary antibodies were purchased from Cell Signaling (Danvers, MA) and used at dilutions of 1:300 and 1:500, respectively. After overnight incubation in primary antibody at 4°C, cells were washed and incubated with AlexaFluor568 (#A11011) at a dilution of 1:2000 for one hour at room temperature. Cells were washed three times in PBS and slides were mounted using VectaShield containing DAPI (#H1200 Vector Laboratories, Burlingame, CA). Fluorescent images were taken at 4000x or 6000x using a Leica DM4000B (Buffalo Grove, IL) upright fluorescent microscope. Leica Application Suite v3.7 (Leica, Buffalo Grove, IL) software was used to capture images. Quantification of c-Jun positive nuclei was performed manually.

### Transient plasmid expression

**AP-1 Transcriptional activity:** MDA-MB-231 cells were seeded to a density of 300,000 cells per 60mm dish and treated with JNK-IN-8 and/or lapatinib. Cells were transfected with 6μg of pGL3-basic.3xAP-1 (Addgene) and 3μg CMV-β-galactosidase. Using a 1:1:1 ratio of DNA:Enhancer™:Lipofectamine 3000®. Lipofectamine/DNA complexes were added dropwise to wells containing the target cells and swirled to mix.

**NFκB and Nrf2 transcriptional activity:** MDA-MB-231 cells and MDA-MB-436 cells were seeded at 100,000 cells/well in 12-well plates and treated with JNK-IN-8 and/or lapatinib. Cells were transfected in serum-free media with 1.25μg of pGL3.5xNFκB-Luc (Promega, Madison, WI) or 1.25μg of NQO1 promoter fused to luciferase gene (a gift from Dr. Dirk Bohmann) and 0.5μg CMV-β-galactosidase. Using a 1:1:1 ratio of DNA:Enhancer™:Lipofectamine 3000®. Lipofectamine/DNA complexes were added dropwise to wells containing the target cells and swirled to mix.

**p65 and Nrf2 expression:** MDA-MB-231 cells were seeded to 3000 cells per well in a 96 well microplate or 175,000 cells per well in 6 well plates. The next day cells were transfected with 200ng (96 well) or 2μg (6 well) of empty vector (pcDNA.3), p65 (RelA cFlag pcDNA3 was a gift from Stephen Smale (Addgene plasmid # 20012)), or Nrf2 (pCDNA3-Myc3-Nrf2 was a gift from Yue Xiong (Addgene plasmid # 21555)). DNA was transfected using *Trans*IT®-BrCa from Mirus Bio LLC (Madison, WI) at 0.4μl or 4μl per well, respectively, according to manufacturers protocol.

### Luciferase assay

Cells were washed twice with cold PBS and lysed using Reporter Lysis Buffer (#E4030 Promega, Madison, WI). Lysate was transferred to black 96 well plates (#3915 Corning, Corning, NY) in triplicate. A BioTek (Winooski, VT) Synergy 2 plate reader with injectors was used to inject 100μL of luciferin in Luciferase Assay Buffer (#E1483, Promega, Madison, WI). Signal was incorporated for 12 seconds immediately after injection and reported as Relative Luciferase Units (RLU).

### β-galactosidase assay

Lysates in Reporter Lysis Buffer were added to clear, round bottom 96 well plates in triplicate. ONPG (o-nitrophenyl-β-D-galactopyranoside) (#369-07-3 Research Products International Corp. Mt. Prospect, IL) substrate was dissolved at 4mg/mL in ONPG buffer (0.120M Na_2_HPO_4_, 0.08M NaH_2_PO_4_, 2mM MgCl_2_, 100mM β-mercaptoethanol) and added to lysates at equal volume. The plate was incubated at 37°C until sufficient yellow color was developed (30 minutes to 1 hour). Absorbance at 420nm was read immediately after injection of 1M sodium carbonate at twice the volume of lysate+ONPG Buffer using the BioTek (Winooski, VT) Synergy 2 plate reader.

### General oxidative stress (ROS) detection assay

Cells were seeded at 2,750 cells/well in black 96-well plates with clear bottoms. After treatment with JNK-IN-8 and/or lapatinib (or H_2_0_2_ for positive control) for the indicated times, DCDFA ROS detection reagent was added to each well to a final concentration of 10μM in phenol red-free media. After a 45 minute incubation in DCDFA, plates were immediately read for fluorescence signal at 495nm/529nm excitation/emission. Fluorescence levels were normalized to cell number by MTT assay. MTT reagent was incubated after fluorescence reading.

### Relative glutathione quantification

MDA-MB-231 and MDA-MB-436 cells were seeded to 40% confluency in white, opaque 96-well plates. After 72 hours of JNK-IN-8 and lapatinib treatment, relative glutathione was quantified according to the manufacturer's instruction using the GSH/GSSG-Glo™ assay (#V6611) from Promega (Madison, WI). Luciferase values were read using the Biotek (Winooski, VT) Synergy2 plate reader.

### Relative NADP^+^/NADPH quantification

MDA-MB-231 and MDA-MB-436 cells were seeded to 40% confluency in white, opaque 96-well plates. After 72 hours of JNK-IN-8 and lapatinib treatment, relative NADP^+^ and NADPH levels were quantified according to the manufacturer's instruction using the NADP^+^/NADPH-Glo™ assay (#G9081) from Promega (Madison, WI). Luciferase values were read using the Biotek (Winooski, VT) Synergy2 plate reader.

## SUPPLEMENTARY MATERIALS FIGURES AND TABLE


