# Supplementary material for: A c-Jun N-terminal kinase inhibitor, JNK-IN-8, sensitizes triple negative breast cancer cells to lapatinib
Source: Oncotarget. 2017 Aug 24;8(62):104894–912. doi: 10.18632/oncotarget.20581 (PMC5739608; doi:10.18632/oncotarget.20581)
Supplement: Supplementary file 1 [file oncotarget-08-104894-s001.pdf]

A c-Jun N-terminal Kinase inhibitor, JNK-IN-8, sensitizes triple negative breast cancer cells to lapatinib

SUPPLEMENTARY MATERIALS

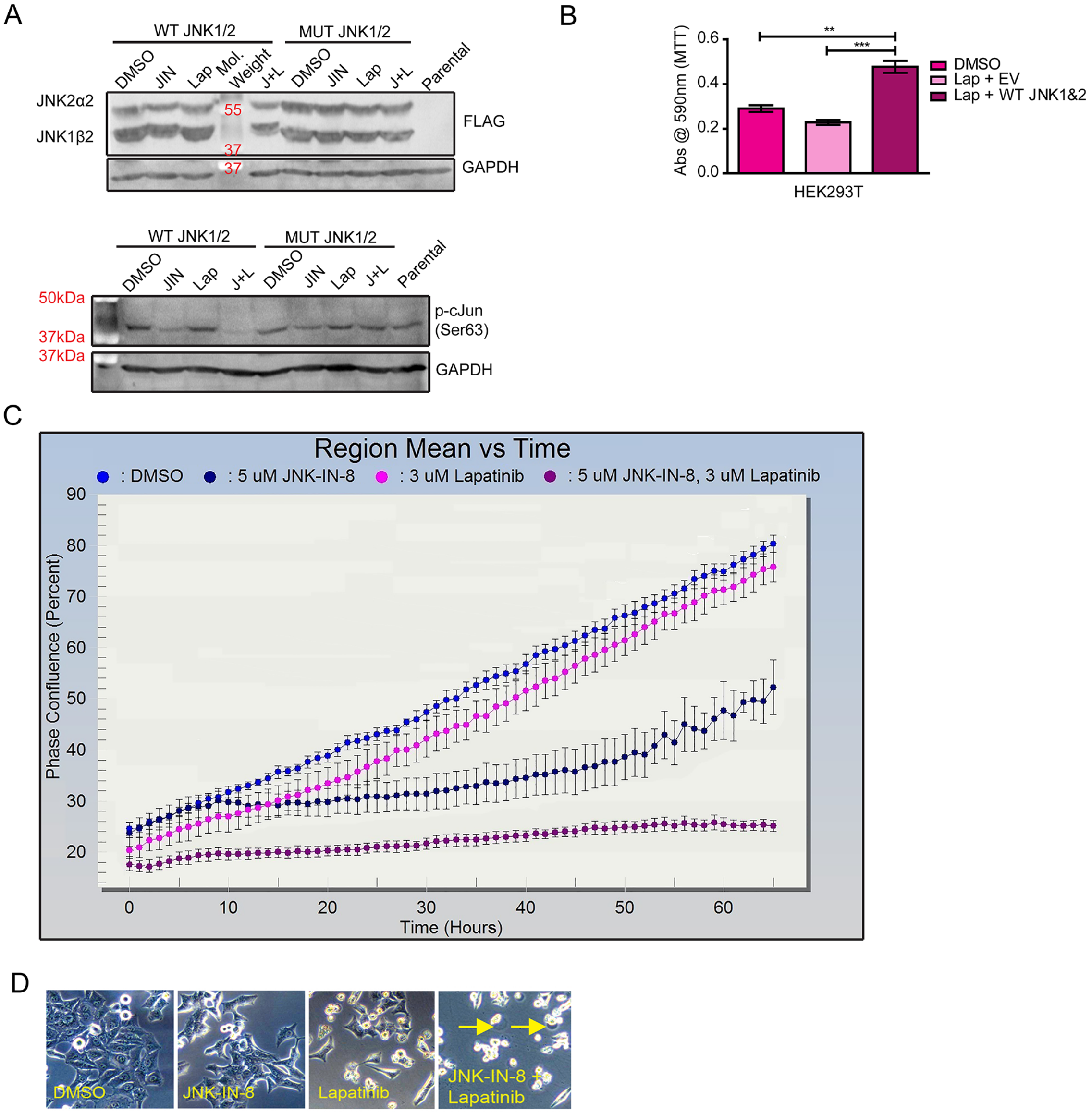

Supplementary Figure 1: JNK-IN-8 Covalently binds JNK through Cysteine 116 and Inhibits c-Jun Phosphorylation. **(A)** HEK-293-T cells were transiently co-transfected with plasmids containing wildtype (WT) JNK1 and JNK2 or JNK1 and JNK2 with a Cys116Ser mutation (MUT). After transfection, cells were treated for 72 hours with vehicle (DMSO), 8μM JNK-IN-8 (JIN), 5μM lapatinib (Lap), or a combination of the two (J+L). Lysates were probed with FLAG, phospho c-Jun (Ser63), and GAPDH (loading control) antibodies. **(B)** Cells transfected with empty vector (EV) or WT JNK plasmids were assayed for viability using MTT after treatment with DMSO or 5μM lapatinib for 72 hours. Raw absorbance values are plotted. **(C)** MDA-MB-231 cells were incubated with vehicle, 5μM JNK-IN-8, and/or 3μM Lapatinib and imaged by phase contrast microscopy using the Incucyte ZOOM every hour. Cell confluency is plotted for 3 wells per treatment. **(D)** Bright field images (1000x) of MDA-MB-231 cells treated with vehicle (DMSO), 5μM JIN, 3μM Lap, or J+L for 72 hours. Arrows indicate membrane blebs.

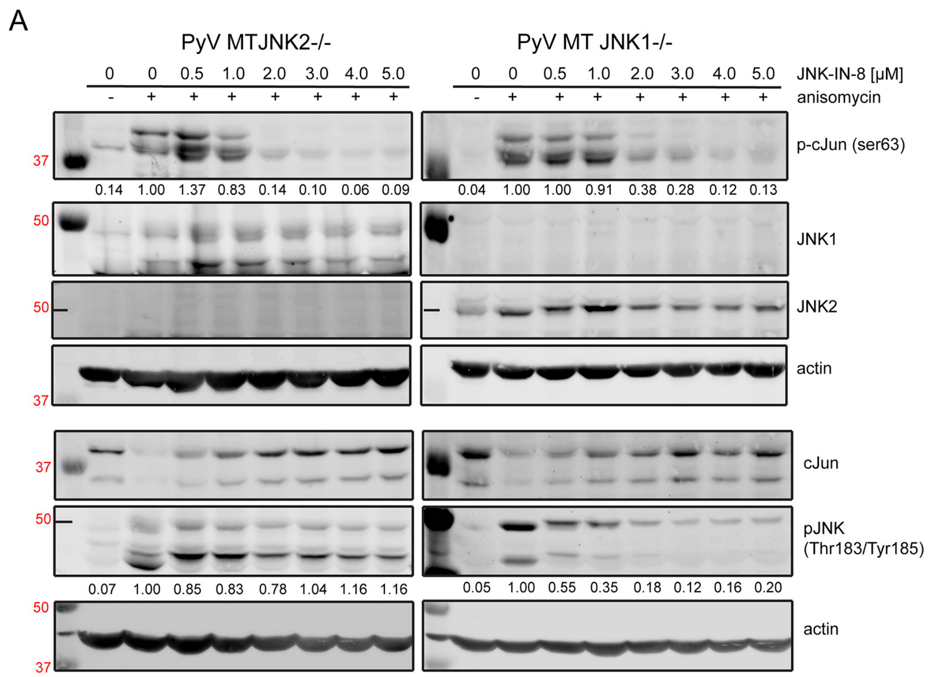

Supplementary Figure 2: JNK-IN-8 similarly inhibits JNK1 and JNK2 isoforms in mammary cancer cells. (A) Cell lines were previously established from mice hemizygous for the Middle-T Antigen and homozygous for JNK2 or JNK1 knockout (PyV MT JNK2-/-, PyV MT JNK1-/-). These cells were serum starved overnight and treated with vehicle or JNK-IN-8 at increasing concentrations for 4 hours. JNK was activated by treatment with anisomycin (8μM) for 45 minutes. Cell lysates were harvested and probed with antibodies against phospho-cJun(ser63), c-Jun, JNK1, JNK2, phospho-JNK (thr183/tyr 185), and actin. Quantifications of phospho c-Jun and phospho-JNK are shown below the panels.

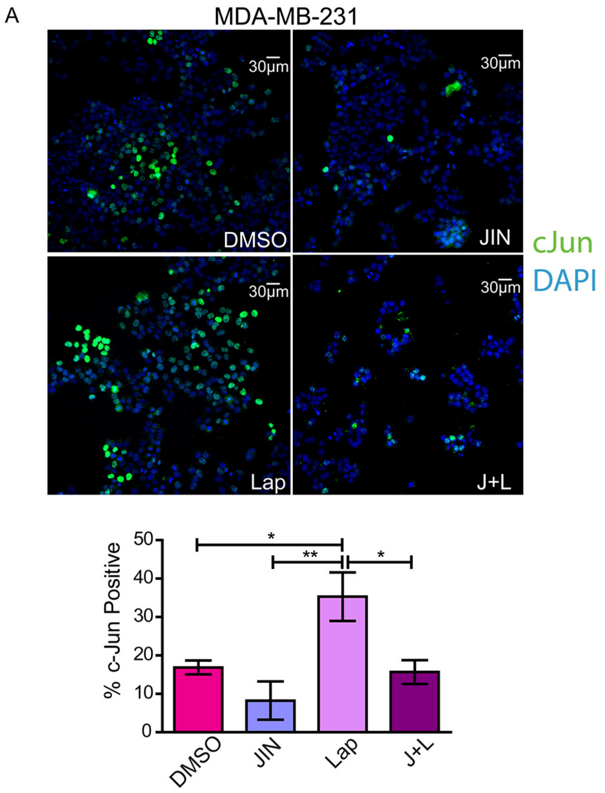

Supplementary Figure 3: Lapatinib Increases c-Jun Expression in Full Serum. (A) MDA-MB-231 cells were treated with vehicle (DMSO), 5μM JNK-IN-8 (JIN) and/or 3μM lapatinib (Lap) for 72 hours. Cells were fixed and stained using immunofluorescence with c-Jun (green) and DAPI (blue). The percentage of c-Jun positive cells out of total is quantified. Bars represent visually counted cells from four separate microscopic fields.

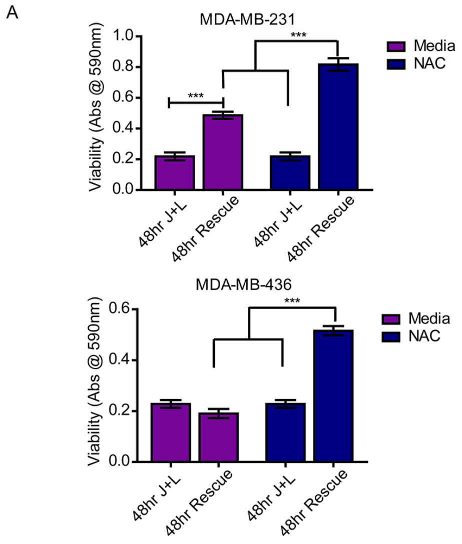

Supplementary Figure 4: Decreased Viability after JNK-IN-8 and Lapatinib Treatment is Rescued by Antioxidants. (A) MDA-MB-231 and MDA-MB-436 cells were treated for 48 hours in 5μM JNK-IN-8 and 3μM lapatinib (J+L) and assayed for viability by MTT. Un-assayed wells were washed twice in PBS and full media or full media + 5mM NAC was added. Viability was assayed 48 hours after by MTT (96 hours after initial treatment).

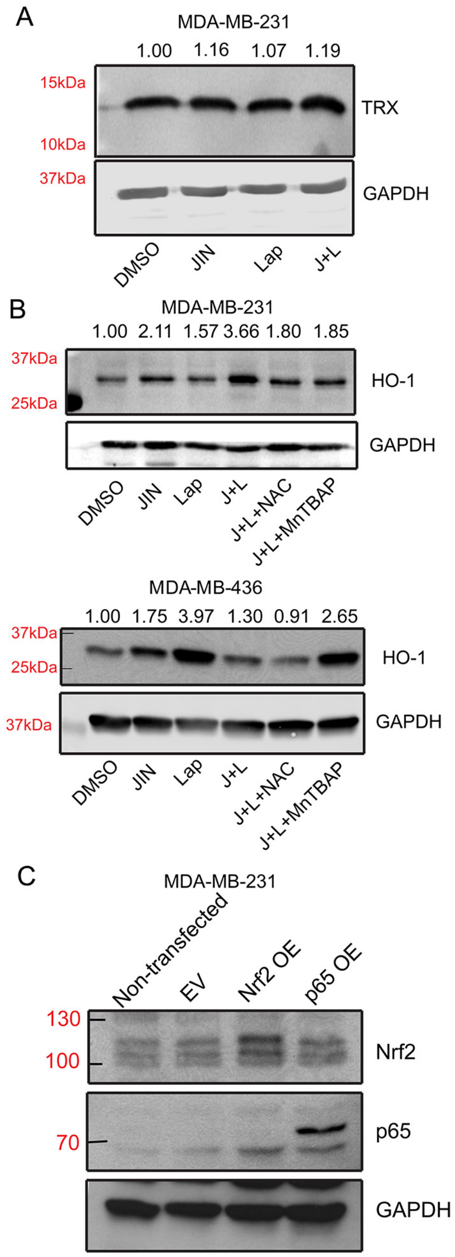

Supplementary Figure 5: Some Nrf2 targets remain unchanged after JNK-IN-8 and lapatinib treatment, however, overexpression of p65 or Nrf2 rescues viability. MDA-MB-231 and MDA-MB-436 cells were treated for 48 hours in DMSO, JIN (3μM), Lap (5μM), J+L, and J+L with NAC (5mM) or MnTBAP (100μM). Lysates were probed for **(A)** thioredoxin and **(B)** HO-1. Densitometries are shown for HO-1. **(C)** MDA-MB-231 cells were non-transfected, transfected with empty vector (EV), or expression plasmids for p65 (p65 OE) and Nrf2 (Nrf2 OE). Forty eight hours after transfection cells were lysed and probed with antibodies against Nrf2, p65, and GAPDH.

Supplementary Table 1: Summary of Phenotypes by Cell Line. For each cell line listed, an "X" is placed in the row where that phenotype was confirmed.

Phenotype	MDA-MB-436	MDA-MB-231	HCC1569	Sk-Br-3
Synergistically decreased viability	X	X	X	X
Increased Apoptosis	X	X	X	
Increased ROS		X		
Viability Rescued by ROS Scavengers	X	X		
Decreased AP-1		X		
Decreased NFκB	X	X		
Decreased Nrf2		X		
Decreased Antioxidants	X	X		
Viability Rescued by p65 or Nrf2 overexpression		X		

X=tested and confirmed, (blank)= not tested
